# A Remote *Cis*-Acting Variant at 3q Links Glomerular NCK1 to Diabetic Nephropathy

**DOI:** 10.1371/journal.pone.0056414

**Published:** 2013-02-18

**Authors:** Bing He, Anne-May Österholm, Juha R. M. Ojala, Ann-Charlotte Andersson, Karl Tryggvason

**Affiliations:** Division of Matrix Biology, Department of Medical Biochemistry and Biophysics, Karolinska Institute, Stockholm, Sweden; University of Louisville, United States of America

## Abstract

We have previously reported genetic association of a single nucleotide polymorphism (SNP), rs1866813, at 3q locus with increased risk of diabetic nephropathy (DN). The SNP is located approximately 70 kb downstream of a cluster of four genes. This raises a question how the remote noncoding polymorphism affects the risk of DN. In this study, we tested a long-range regulatory potential of this variant by a series of experiments. In a luciferase assay, two alleles of the SNP showed differential effects on the luciferase activity in transfected cells *in vitro*. Using transgenic zebrafish, we further demonstrated *in vivo* that two alleles of the SNP differentially regulated GFP expression in zebrafish podocytes. Immunofluorescence staining and Western blotting verified that only *Nck1* of the four nearby genes was predominantly expressed in mouse glomeruli as well as in podocytes. Furthermore, genotypes of the SNP rs1866813 were correlated with *NCK1* expression in immortalized lymphocytes from diabetic patients. The risk allele was associated with increased *NCK1* expression compared to the non-risk allele, consistent with the results of the reporter-based studies. Interestingly, differential expression of glomerular *Nck1* between mouse strains carrying the nephropathy-prone 129/Sv allele and nephropathy-resistant C57BL/6 allele was also observed. Our results show that the DN-associated SNP rs1866813 is a remote *cis*-acting variant differentially regulating glomerular *NCK1* expression. This finding implicates an important role for glomerular NCK1 in DN pathogenesis under hyperglycemia.

## Introduction

Diabetic nephropathy (DN) is the leading cause of end-stage renal disease (ESRD) [1]. Clinically, DN is characterized by progressive proteinuria, relentless decline in kidney function accompanied by arterial hypertension, and increased risk of cardiovascular disease [1], [2]. DN primarily affects the kidney glomeruli and manifests pathologically as thickening of the glomerular basement membrane (GBM), with consequent mesangial expansion and glomerular sclerosis [2]. Proteinuria in DN is mainly due to disturbances in the glomerular filtration barrier that is composed of three main layers: fenestrated endothelial cells, GBM and podocytes with their interdigitating foot processes and slit diaphragm located between them [2], [3], [4], [5]. However, the underlying mechanisms of DN still remain poorly understood.

It has been extensively documented that hyperglycemia cause changes in cell metabolism, glycosylation and long-term modification of extracellular proteins and consequently endothelial and podocyte damage and glomerular injury [6]. A recent study shows that even normoalbuminuria in type 2 diabetic patients bear significant cardiovascular risk [7]. It is also well known that only about one-third of the diabetic patients develop nephropathy due to hyperglycemia [1]. The majority of diabetic patients apparently do not have genetic susceptibility to develop hyperglycemia-driven microangiopathy and, therefore, there has thus far been limited success in identifying susceptibility genes for DN [8], [9]. Using genome-wide linkage analyses in siblings, the chromosome 3q locus has been linked to DN in four independent populations, *i.e.* Pima Indians [10], Caucasian Americans [11], African Americans [12] as well as Finns [13]. Based on the fine linkage mapping of the 3q locus [13], we have recently identified a DN-associated SNP, rs1866813, with a combined odds ratio of 1.33 [14]. This variant resides in an 11-kb high linkage disequilibrium (LD) region, 70 kb downstream of a cluster of four genes (*STAG1*, *TMEM22*, *NCK1* and *IL-20RB*). Of those genes, mouse *Nck1* has been associated with the formation of foot processes during podocyte development in mice [15] and regeneration following glomerular injury [16]. However, the importance of human *NCK1* in DN etiology is unknown. Therefore, it raises important questions whether this remote noncoding DNA polymorphism is functional, and furthermore how it could affect DN risk.

Despite the fact that genome-wide association studies have identified a number of SNPs associated with complex diseases, *in vivo* functional validation of these SNPs in influencing disease risk has been sparsely reported [17], [18], [19], [20]. The *cis*-regulatory elements including enhancers and silencers usually function over kilobase- or even megabase-long genomic distances to modulate target gene expression patterns [21]. Accordingly, we experimentally followed up our association findings under a hypothesis that the rs1866813 lies in a *cis*-acting sequence directing an allele-related expression of one or more of the nearby genes through a long-range regulatory mechanism. In this study, we demonstrate that the DN-associated SNP rs1866813 differentially regulate reporter gene expression in transfected cells and transgenic zebrafish podocytes. Moreover, we verify that mouse *Nck1* is predominantly expressed in glomerulus and genotype of rs1866813 is correlated with human *NCK1* expression levels, supporting that glomerular *NCK1* expression is modulated by the variant. Together, these results provide evidence of a connection between glomerular NCK1 and DN and implicate an important role for glomerular NCK1 in DN pathogenesis under hyperglycemia.

## Results

### In Vitro Luciferase Assay

**Figure 1:**
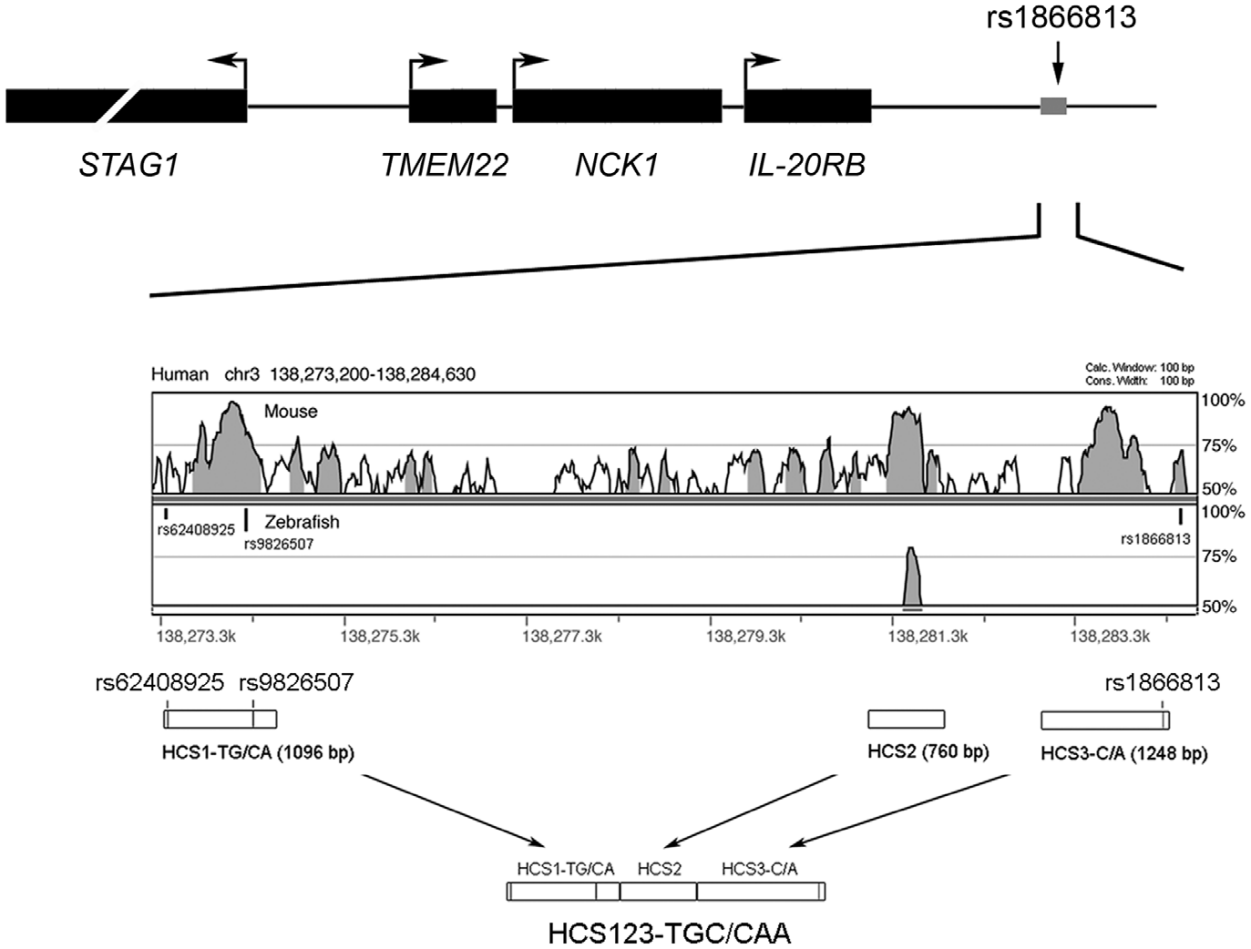
Genomic conservation analysis. The upper panel illustrates the physical map of the SNP rs1866813 and the four nearby genes. The 11-kb high LD block containing rs1866813 is indicated with a gray box. The VISTA plot displays that three human conserved sequences (HCS1-3) are highly conserved (score >90%) against mouse genome and one sequence is aligned with orthologous zebrafish region (score >75%). The position of three SNPs (rs62408925, rs9826507 and rs1866813) is indicated with vertical black bars. The lower panel illustrates generation of the constructs used for functional analyses. Open boxes denote PCR amplicons of HCS1-3 with SNPs and size of the amplicons is indicated in parenthesis. Three single HCSs were ligated together leading to two combined haplotype constructs, HCS123-TGC and -CAA, indicated with three long arrows.

Our previous study showed that the SNP rs1866813 lies in a high LD region containing three human conserved sequences (HCS1-3) [14]. Using the online program VISTA Browser, we evaluated quantitatively conservation scores of the 11-kb rs1866813-carrying region flanked by rs62408925 and rs1866813, among human, mouse and zebrafish (Figure 1). We confirmed that HCS1, 2 and 3 were highly conserved compared with the mouse sequence with scores of >90%, and that rs1866813 is located in the vicinity of HCS3 (Figure 1). However, HCS1 and 3 showed no conserved signals against the zebrafish sequence, though HCS2 without variations was weakly conserved with a score of around 75% (Figure 1).

**Figure 2:**
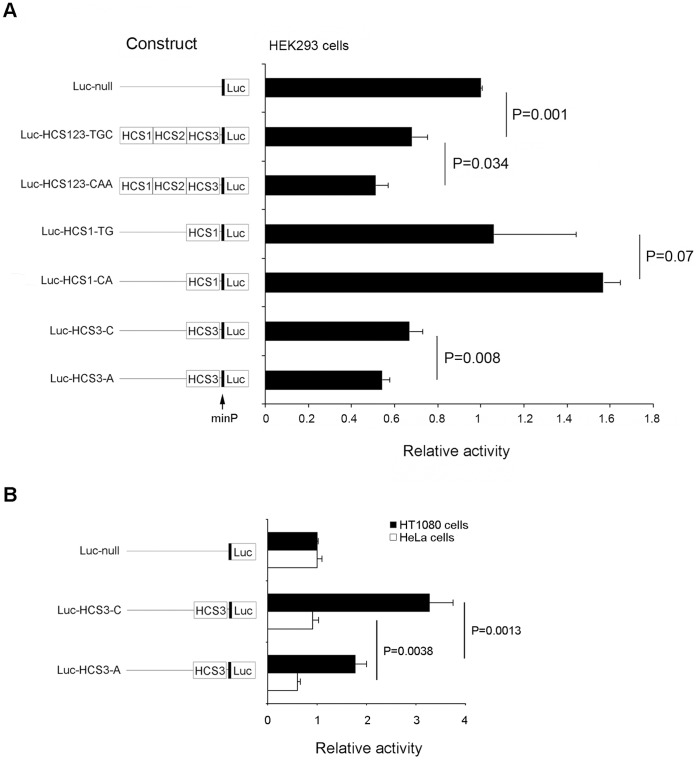
Luciferase activity. (A) Luciferase reporter constructs with different inserts were transiently transfected in HEK293 cells. (B) The construct with HCS3-C or HCS3-A was transiently transfected in HT1080 or HeLa cells. Relative luciferase activity was obtained by setting the relative luciferase activity of the empty plasmid (Luc-null) to be 1. Schematic illustration of luciferase constructs used for the assays are shown on the left side of the bar graph. A minimal promoter (minP) is indicated with an arrow.

To test *cis*-acting potentials of the conserved elements, we first used a luciferase expression system. The plasmids with different individual HCSs and haplotype elements, illustrated in Figure 1, were transiently transfected in human embryonic kidney cells (HEK293). As shown in Figure 2A, luciferase relative activity driven by a plasmid with the non-risk CAA haplotype (HCS1-3) was 25% lower than the at-risk TGC haplotype (*P*  = 0.034), suggesting an allele-related expression pattern. A similar pattern was consistently observed with the non-risk allele A in HCS3 leading to 18% lower activity than the risk allele C (*P*  = 0.008). However, TG and CA haplotypes in HCS1 did not lead to significant difference in luciferase activity (*P*  = 0.07). The different *cis*-effects of HCS123 haplotypes are likely controlled by the SNP rs1866813. Therefore, we only used rs1866813-carrying HCS3 in subsequent experiments. To substantiate this finding, we further transfected the risk- and non-risk allele constructs HCS3-C and -A in HeLa cells and human fibrosarcoma cells (HT1080), respectively. Again, similar patterns showing significant difference in activity between the risk allele C and non-risk allele A were found in Figure 2B. The C allele shows a gain-of-function compared with the A allele regardless of baselines in different cell types. Together, these results indicate that rs1866813 in HCS3 is a *cis*-acting variant and that the risk C allele drives higher expression activity compared with the non-risk A allele in the cell culture system.

### *In Vivo* Transgenic Zebrafish Study

**Figure 3:**
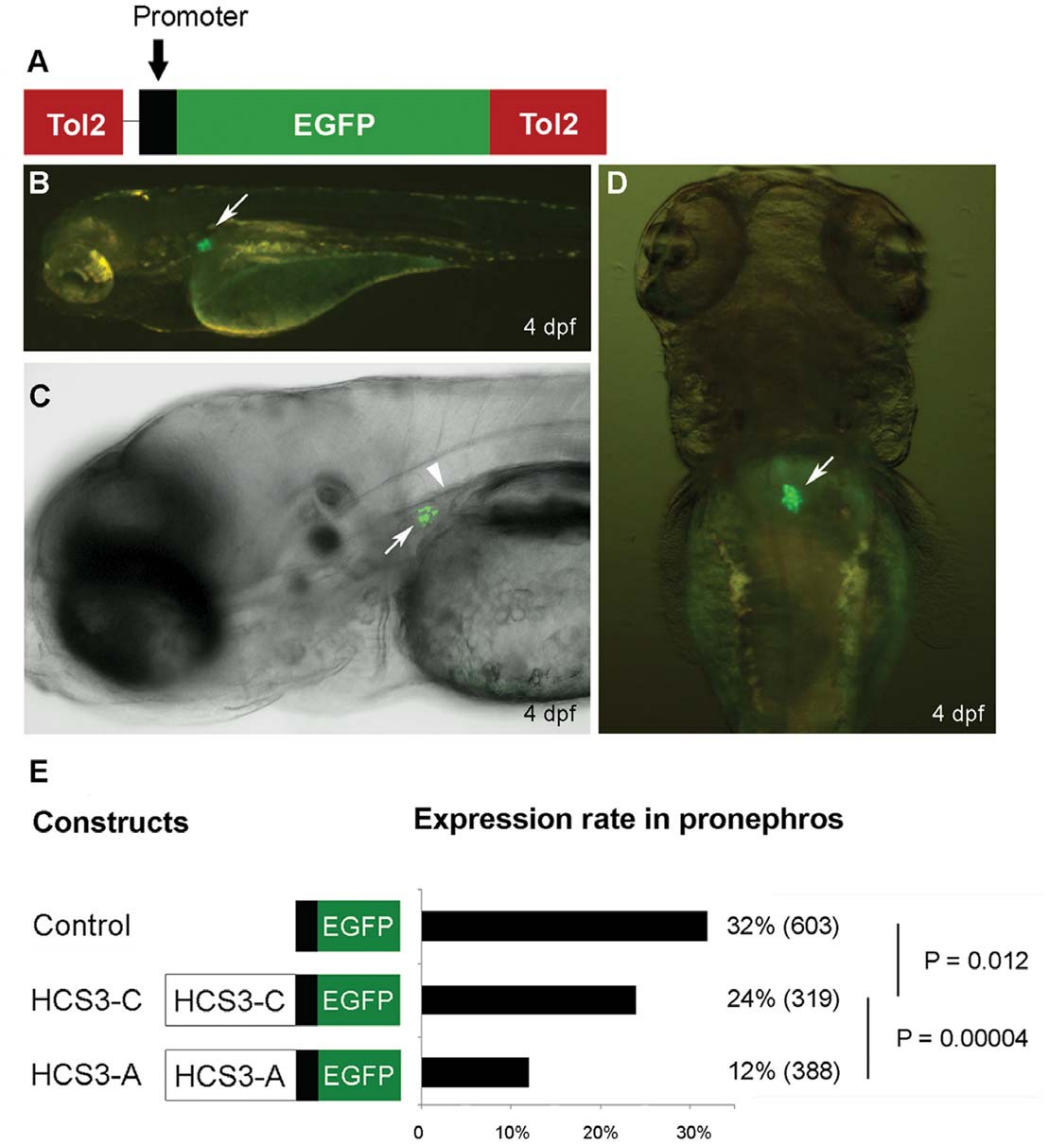
Transgenic zebrafish study. (A) Schematic view of the Tol2 transposon-based plasmid carrying a 185 bp zebrafish *nphs2* promoter. (B-D) Transient GFP expression in zebrafish podocytes at 4 dpf. Lateral view (B), confocal image (C), and dorsal view (D). Pronephros and dorsal aorta are indicated by arrows and an arrowhead, respectively. (E) GFP expression rate in 4 dpf-embryos injected with different constructs. The plasmid without inserts was used as a control for the baseline. The HCS3-A or -C sequence was subcloned upstream of the promoter. They are schematically shown in left panel. The bar graph illustrates expression rate (%) and total number of G_0_ embryos for assessment are indicated in parentheses.

We next asked whether SNP rs1866813 results in an allelic expression of the gene(s) in kidney podocytes *in vivo*. Recently, we have established an *in vivo* transient podocyte expression system, in which the Tol2-based plasmid contains a short zebrafish *nphs2* (podocin) promoter that robustly drives GFP expression in the pronephric podocytes in injected G_0_ embryos (Figure 3A, B, C, D, E) (unpublished data). This system may be specially suited for evaluation of elements with podocyte-specific regulatory potentials. Using this system, we found that, in control group, 32% of injected embryos showed GFP expression in podocytes. In test groups, the two alleles of rs1866813 differentially drove GFP expression, in which allele A led to 12% expression and allele C to 24% (*P*<0.0001) (Figure 3E). In agreement with results of *in vitro* luciferase assays, the C allele in podocytes *in vivo* upregulated GFP expression in comparison with the A allele as a reference. Thus, the human rs1866813-carrying HCS3 also exerts differential regulatory functions on gene expression in living zebrafish podocytes, suggesting conserved regulatory mechanisms among vertebrates.

### Glomerular Expression of the Four Nearby Genes

**Figure 4:**
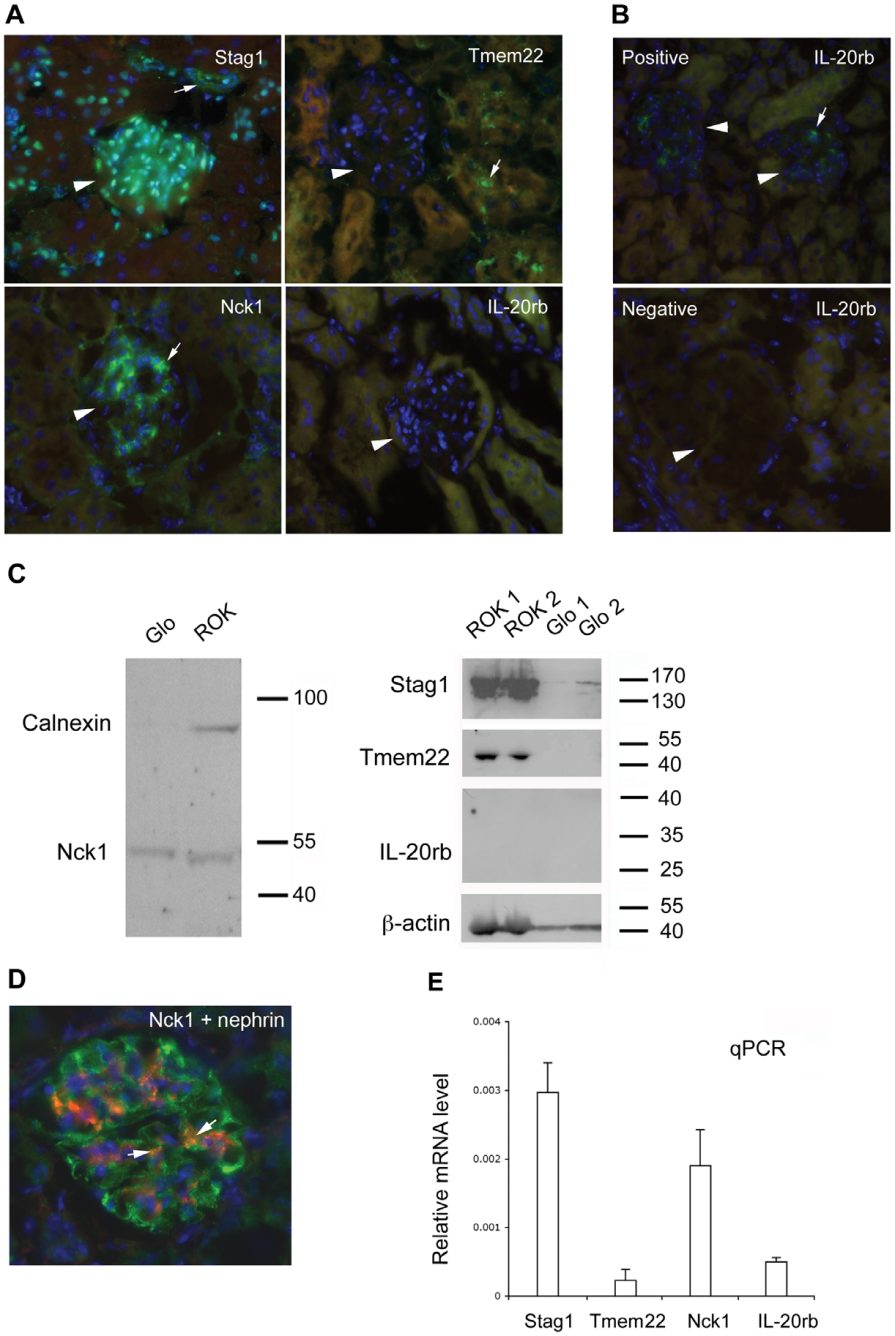
Glomerular expression analysis of four nearby genes. (A) Immunofluorescence staining of mouse kidney sections. Positive signals of staining and locations of glomeruli are indicated by arrows and arrowheads, respectively. (B) Positive and negative controls of mouse kidney immunofluorescence staining for IL-20rb. LPS-treated mouse kidney was used as a positive control. Staining without primary antibody to IL-20rb was used as a negative control. (C) Western blotting analysis. Nck1 and calnexin, an internal control, are shown in the left side. Stag1, Tmem22, IL-20rb and β-action, an internal control, are shown in the right side. Glo, the glomerular lysate; ROK, the rest of kidney, indicating lysates from kidney that lacks glomeruli fractions. Molecular weight is indicated by number of kDa. (D) Double immunostaining of mouse kidney sections with a podocyte marker nephrin (green) and Nck1 (red). Yellow color pointed by arrows indicates partial colocaliztion of staining of Nck1 and nephrin staining. (E) qPCR analysis. mRNA expression of four genes from isolated glomeruli of adult C57BL/6 mouse kidney was quantified using the TaqMan method.

Since kidney glomerulus is the primarily affected target in DN, the candidate gene(s) important for DN should be expressed mainly in the glomeruli. Therefore, the glomerular expression was used as the criteria to determine which of the four nearby genes (*STAG1*, *TMEM22*, *NCK1* and *IL-20RB*) is potentially regulated by the SNP. As shown in Figure 4A, using immunofluorescence stainings of normal mouse kidneys, Tmem22 expression could be detected in the tubular area, but glomerular staining was completely absent. Similarly, IL-20rb staining was absent in the normal kidney, but could be detected in LPS-treated mouse kidney glomeruli (Figure 4B). These results were further confirmed by Western blotting, in which lysates from glomerular fractions were negative for Tmem22 and IL-20rb (Figure 4C). Stag1, a nuclear protein [22], was ubiquitously expressed in all renal cellular nuclei including tubules, ducts and glomeruli (Figure 4A). Western blotting showed strong signals of Stag1 in lysates from non-glomerular fractions (rest of kidney) (Figure 4C), suggesting that it is unlikely to be a real DN-associated protein. Both immunofluorescence and Western blotting illustrated that, out of the tested four candidate genes, only Nck1 was predominantly present in glomeruli (Figure 4A, C). To verify whether Nck1 is present in glomerular podocytes, we performed double staining with antibodies to Nck1 and to nephrin (a podocyte marker). In Figure 4D, overlapping staining for Nck1 and nephrin in the mouse glomerulus was visible, supporting Nck1 expression in podocytes. As reported earlier, additional Nck1 staining was also detected in mesangial cells [15]. We also examined transcripts of the four genes in isolated mouse glomeruli, confirming a high transcription level of *Nck1* in comparison with Tmem22 and IL-20rb (Figure 4E). These results thus support Nck1 as a good DN candidate probably targeted by the remote *cis*-regulatory variant.

### NCK1 Expression and Genotypes of rs1866813

**Figure 5:**
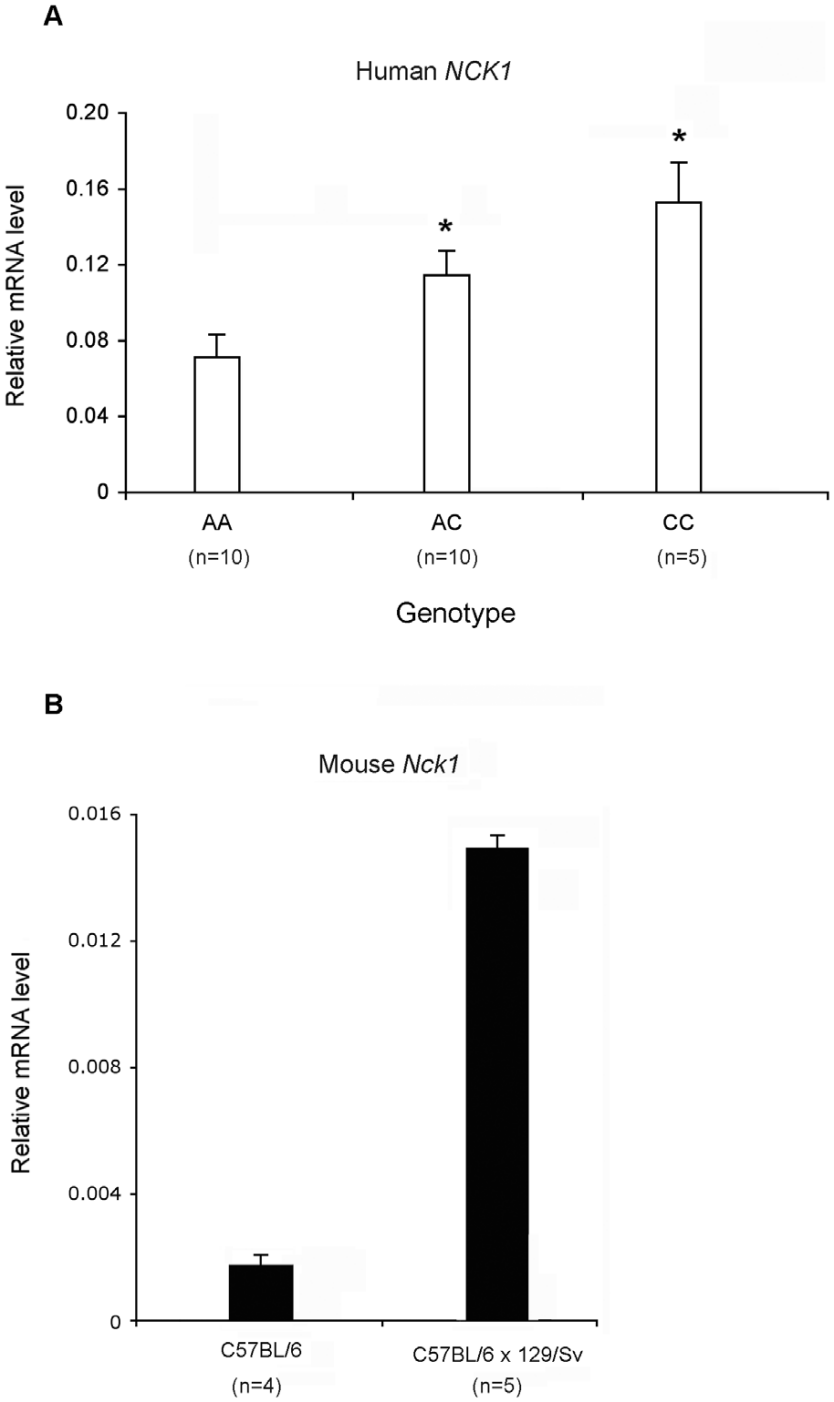
Differential expression of *NCK1* in humans and mice. (A) Correlation of *NCK1* expression with genotypes of the SNP rs1866813. Relative *NCK1* mRNA levels in three genotype groups are shown in open bars. Total RNA was extracted from immortalized lymphocytes derived from 25 diabetic patients with different rs1866813 genotypes and their numbers are indicated in parentheses. These cells were cultured under 5 mM glucose. (B) Differential expression of glomerular *Nck1* in mice with different strains. Relative *Nck1* mRNA levels in isolated glomeruli from four C57BL/6 mice and five mice with C57BL/6×129/Sv background are shown in a black bar. Bars represent mean ± s.e.m 2^-ΔCt^. *P<0.05.

To validate if *NCK1* expression is modulated by the rs1866813, we then analyzed the relationship between expression levels of endogenous *NCK1* and genotypes of the SNP. This was done on total RNA extracted from immortalized human lymphocytes derived from 25 diabetic patients with genotype data of the rs1866813. Among them, 15 patients were diagnosed as DN with macroalbuminuira or ESRD and 10 patients were normoalbuminuric over at least 15 years after diagnosis of diabetes. *NCK1* expression level was quantified by qPCR. As shown in Figure 5A, under normal glucose conditions, *NCK1* expression was correlated with genotypes of rs1866813, in which *NCK1* expression was upregulated in both AC and CC carriers in comparison with its expression in AA carriers (*P*<0.05). *NCK1* expression in CC carriers (n = 5) was higher than that in AC carriers (n = 10), but the difference between the two groups did not reach statistical significance (*P*  = 0.12). We further tested for correlation of clinical characteristics including diabetic duration, levels of glycosylated hemoglobin (HbA1c) and DN with the C-allele carriers (AC+CC) in this small sample set. In Table 1, we did not find significant correlations of the medical status with genotypes (*P*>0.05 for all comparisons), though DN showed a trend in association with the C-allele carriers.

**Table 1:** Correlation analysis of clinical characteristics of 25 diabetic patients with their genotypes of the SNP rs1866813.

Genotype	n	Diabetic duration (years)	DN	HbA1c (%)	Blood pressure (mmHg)Systolic Diastolic	
AA	10	30.63±8.58	4 (40%)	10.32±1.97	133.60±9.71	83.20±7.36
AC+CC	15	31.23±7.18	11 (73%)	8.54±1.71	144.00±19.92	80.86±8.59
P value		0.87	0.21	0.14	0.26	0.62

We then tested whether differential expression of glomerular NCK1 identified in humans is present in mouse strains. Interestingly, *Nck1* glomerular expression in mice with a nephropathy-prone mixed background of C57BL/6 and 129/Sv was almost 10-fold higher than that in nephropathy-resistant C57BL/6 mice (Figure 5B). The allele from 129/Sv mice most likely contributes to the increased *Nck1* expression in glomeruli. Thus, differential glomerular expression of *Nck1* also exists in different mouse strains.

## Discussion

Like many complex disease-associated SNPs identified by genome-wide association studies, the rs1866813 resides in an intergenic noncoding region. Thus, functional validation of identified DNA polymorphisms contributing to the DN pathogenesis has been challenging. In this study, we experimentally validate the regulatory importance of the DN-associated SNP rs1866813 and show that its two alleles differentially regulate glomerular *NCK1* expression through a long-range regulatory mechanism. Our finding suggests a possible role for glomerular NCK1 in the pathogenesis of nephropathy in patients with diabetes.

DNA sequences conserved among multiple species may imply a functional potential [23]. Therefore, we first analyzed whether rs1866813 resides in an evolutionarily conserved region. High conservation of the rs1866813-carrying region across mammals suggests a regulatory potential and prompted us to perform *in vitro* experiments for the initial functional validation. Due to high LD of rs1866813-carrying region, three conserved elements were subcloned together, resulting in two haplotype constructs, TGC and CAA. The idea was to test whether a *cis*-acting effect differs between combined haplotypes and single elements. Using *in vitro* luciferase assays, activity driven by haplotype constructs showed a pattern similar to that by the single rs1866813-carrying constructs. This suggests that the rs1866813-carrying HCS3 element harbors a *cis*-regulatory element controlling the entire haplotype region, since the polymorphisms in HCS1 did not show significant allelic effects on luciferase activity. This result is also supported by our previous association study, where rs1866813 shows the strongest association with DN among any other LD-correlated SNPs in the region [14]. The *cis*-effect by allele A or C of rs1866813 on reporter activity is consistently and significantly different in three different transfected cell lines, supporting the notion of the *cis*-acting variant. Moreover, the risk C allele consistently results in higher luciferase expression than the non-risk A allele in all tested cells. Meanwhile, we observed that *cis*-effects of the HCS haplotypes and HCS3s on luciferase activities in different cell lines differ; for example, both HCS haplotyeps and HCS3s showed a repressive effect in HEK-293 cells, but an enhancing effect in HT1080 cells when compared to the basal level of luciferase activity (Figure 2A, B). Supporting our findings, a similar phenomenon has been reported for C950T SNP-carrying element, which had a repressive effect on luciferase activities in HeLa cells, however, an enhancing effect in COS-7 cells [24]. It is possible that basal transcription activities on the *cis*-regulatory elements analyzed in different cell lines significantly differ, though precise mechanisms remain to be clarified.

The *cis*-acting sequence exerts its regulatory action through binding of transcription factors in some cells where they are expressed. Therefore, it is important to provide evidence whether the rs1866813-carrying HCS3 exerts a *cis*-regulatory effect in glomerular podocytes *in vivo*. Podocytes are terminally differentiated cells, and they undergo a rapid dedifferentiation under cultured conditions [3]. These features indicate that cultured podocytes quite poorly mimic the complex characteristics of *in vivo* podocytes. To address this critical concern, we analyzed the HCS3 *in vivo* using a transgenic zebrafish model. Zebrafish embryos possess a simple pronephros that functionally accomplishes blood filtration and osmoregulation at embryonic and larval stages [25]. The pronephric kidney resembles structurally and functionally mammalian metanephric kidney [26]. In particular, pronephric glomerulus in zebrafish displays a fine architecture of interdigitating foot processes, similar to the mammalian counterparts, under the electron microscope after 4 days post fertilization (dpf) [25], [27], [28]. Based on our recently generated zebrafish podocin-GFP line [28], we validated that transient GFP expression driven by the 2.5 kb zebrafish *nphs2* promoter accurately reflects expression in the germline zebrafish. Then we further narrowed this 2.5 kb promoter fragment into 185 bp upstream of the transcription start site, which was sufficient to direct robust GFP expression in zebrafish podocytes (Unpublished data). This system allows a rapid evaluation of potential *cis*-acting sequences, in particular, sequences with negative regulatory effects in podocytes. Other screening tools commonly used for assessing *cis*-acting potentials in fish are only designed for enhancers [29]. Using this *in vivo* expression system, we found that two alleles of rs1866813 in HCS3 can differentially regulate GFP expression in zebrafish podocytes and again, the C allele upregulated GFP expression when compared to the A allele. Though HCS3 element does not show a significant conservation score against zebrafish genomic sequence (Figure 1), its influence on the podocyte promoter indicates a conserved regulatory mechanism among vertebrates. Our results confirm previously published data, where human sequence flanking the RET gene, conserved across rodents, can drive GFP expression in zebrafish target cells, though there is no sequence similarity between human and zebrafish [30].

A cluster of four genes (*STAG1, TMEM22, NCK1* and *IL-20RB*) are distantly located upstream of the SNP rs1866813. Theoretically, all these genes could be regulated by the rs1866813-carrying element. We reasoned that the target gene(s) affected by the DN risk allele should be expressed mainly in the glomerulus and some of these four genes are preferentially modulated by the rs1866813-carrying element in glomerular podocytes. Therefore, we performed immunofluorescence staining and Western blotting as well as qPCR to examine expression distribution of four nearby genes in kidney from adult wild-type C57BL/6 mice. Consistent results detected by the three methods verified the absence of Tmem22 and IL-20rb in glomerulus. Stag1 is excluded due to the fact that it is mainly expressed in the non-glomerular portion of kidney. These results support that only Nck1 is predominantly expressed in glomerulus.

We further show that genotypes of rs1866813 are correlated with endogenous *NCK1* expression levels in cultured cell lines derived from diabetic patients, in which the risk C allele is associated with increased *NCK1* transcript levels, compared with the non-risk A allele. The effect of the C-allele on *NCK1* expression shows an additive trend (Figure 5A), suggesting an allele dosage function. The fact that *NCK1* expression between CC- and AC-carriers does not reach statistical significance may be due to too few individuals with the risk CC allele included for analysis. The small size of samples may also lead to a low power to find a significant correlation of clinical characteristics with the C-allele of rs1866813. Taken together, these results provided experimental evidence linking glomerular NCK1, a well-characterized slit diaphragm-associated protein, to DN. NCK is an adapter protein and it has been shown in mouse experiments a crucial link between phosphorylated nephrin and the actin cytoskeleton during development of foot processes, as well as in their regeneration during repair of effaced foot processes after glomerular injury [15], [16]. Furthermore, Nck proteins are also required for maintenance of the adult glomerular filtration barrier [31]. Besides the nephrin-Nck pathway, Crk1/2-dependent signaling has also been shown necessary for mediating nephrin-directed cytoskeletal dynamics in the podocyte [32]. In contrast to other complex diseases, gene products targeted by DN risk allele(s) appear to be physiologically normal until long exposure to hyperglycemia. However, little is known about relationship between hyperglycemia and NCK1. Thus, hyperglycemic impact on glomerular NCK1 or its transcription activity requires further studies.

Mouse models of DN have been widely used for identification of specific factors or to predict DN. It is generally accepted that C57BL/6 mouse is the most resistant to diabetic nephropathy among tested strains [33], while 129/Sv mouse is prone to renal injury [34]. In this study, we also analyzed glomerular *Nck1* expression in mice with different genetic backgrounds. Interestingly, differential expression of glomerular *Nck1* exists in these two mouse strains. Glomerular *Nck1* expression in 126/Sv allele-carrying mice is 10-fold higher than that in C57BL/6 mice. Whether mice with high Nck1 in podocytes are more susceptible to kidney injury than those with low Nck1 remains to be clarified.

In summary, we demonstrate the functional importance of the DN-associated variant rs1866813 based on different experimental systems. The risk allele contributes to significant upregulation of glomerular *NCK1* expression in comparison with the non-risk allele. Our finding suggests a new role for glomerular NCK1 in the pathogenesis of DN, and provides an important clue for further diabetic animal studies.

## Materials and Methods

### Comparative Sequence Analysis

The VISTA Browser (http://genome.lbl.gov/vista) was used to examine genomic conservation of the 11-kb sequence carrying rs1866813 across mouse and zebrafish whole genome assemblies. The conservation score from 75% to 100% was displayed by this method. Conservation was measured using a 100-bp window and a cut-off score of 50% identity.

### Plasmids and Cloning

To evaluate *cis*-regulatory potentials of three HCSs with SNPs *in vitro* and *in vivo*, DNA sequences were first amplified from two human samples with TGC or CAA haplotype. Primer sequences used to amplify HCS1-3 from human genomic DNA are as follows: HCS1∶5′-TTCCCACTGGGTTCTGTTAG, 5′-GGATCCGGCTGCTGCATTTTCTGAAC; HCS2∶5′-CATGGTAGGCCTAAACATCC, 5′-ATTTGGCTCCCCATGTACTC; HCS3∶5′-GCGGCCGCCATACTTGGCAGATCACTGG, 5′-CGTAAGCCCAGCATCATGTA. An annealing temperature of 55°C was used for all the reactions. The HCS1 carrying two SNPs (rs62408925 and rs9826507) led to two amplicons with TG or CA haplotypes. The HCS2 has no polymorphisms. The HCS3 with a single SNP rs1866813 led to two amplicons with a C or A allele. These five amplicons were cloned into pCRII-TOPO vectors (Invitrogen), respectively. To test potentials of *cis* effects of the three-SNP haplotypes, TGC or CAA, three individual HCSs were ligated together, resulting in two HCS123-containing constructs with TGC or CAA haplotype (Figure 1). These cloned DNA sequences confirmed by sequencing were then subcloned into either the luciferase pGL4.26 plasmid with a minimal promoter between SacI and XhoI sites (Promega) or the Tol2-based plasmids described below. An empty pGL4.26 plasmid (Luc-null) and empty Tol2-based plasmid without inserts were used for the baseline, illustrated schematically in Figure 2A, B and Figure 3A, E.

### Cell Culture

In this study, three commercially purchased cell lines were used; they were the human embryonic kidney cell line (HEK293) (ATCC No. VR-681), the HeLa cell line, derived from human cervical cancer, (ATCC No. CCL-2), and the human fibrosarcoma cell line (HT1080) (ATCC No. CCL-121). Cells were cultured in standard DMEM media. The EB virus-transformed primary human lymphocytes, derived from 25 diabetic patients with different genotypes of rs1866813 (AA = 10, CA = 10 and CC = 5), were used for the qPCR analysis. The main clinical data of these 25 patients with their genotypes are summarized in Table 1. The lymphocytes cultured in the RPMI-1640 medium were seeded onto a six-well plate with 10^6^ cells in 1.5 ml/well. Cells were incubated in a starvation medium containing 2.5 mM glucose and 1% fetal calf serum (FCS) prior to the start of the experiment. After this period, we adjusted FCS to 10% and glucose to 5 or 25 mM. The cells were harvested after one-week exposure to glucose for total RNA extraction.

### Luciferase Assay

One day before transfection, 10^5^ cells in 0.5 ml were seeded in 24-well plates. Then, 0.5 µg of the pGL4.26 constructs were co-transfected together with 0.01 µg of the pRL-TK Renilla luciferase vector (Promega) as an internal control, using 0.5 µl of Lipofectamin 2000 reagent (Invitrogen). Quadruplicate transfections for each sample were performed. After 24 h, luciferase activity assayed using the Dual-Luciferase Reporter Assay System (Promega) was normalized by the pRL-TK reporter activity. Relative activity values represented luciferase activity relative to the baseline of normalized luciferase activity from the Luc-null plasmid, which was arbitrarily set to 1. Data was presented as mean ± SD.

### Transgenic Zebrafish

We modified the previously used Tol2 transposon-based plasmid carrying a 2.5 kb zebrafish *nphs2* promoter [28]. Our recent study showed that the 185 bp *nphs2* promoter was sufficient to direct GFP expression in pronephric podocytes (Unpublished data). The HCS3-C and -A sequences were inserted upstream of the short promoter (Figure 3A), respectively. The constructs, together with the Tol2 transposase mRNA, were co-injected into 1 to 2-cell AB strain zebrafish embryos as previously described [28], [35]. We analyzed transient GFP expression in at least 200 injected G_0_ embryos at 4 dpf under the fluorescence microscope (Leica). An embryo showing GFP expression in pronephric glomerulus (Figure 3B–D) was evaluated as a positive one. The positive rate (%) was presented.

### Immunofluorescence Staining and Western Blotting

The kidney from adult C57BL/6 mice was snap-frozen and embedded in OCT. Cryosections (8 µm) were postfixed with cold acetone for 10 min followed by blocking in 5% normal goat or donkey serum. The primary antibodies Stag1 (1∶100, Santa Cruz, goat), Tmem22 (1∶100, Santa Cruz, rabbit), Nck1 (1∶250, Abcam, rabbit monoclonal), IL-20rb (1∶100, Abcam, rat) and nephrin (1∶200, Acris GmbH, guinea pig) were incubated at 37°C for 1 h, followed by 45 min incubation with corresponding Alexa fluor (Invitrogen) secondary antibodies. To validate primary antibody to IL-20rb, kidney from LPS-treated mice was used. Negative controls without primary antibodies were performed and showed no signals for all four antibodies. Western blotting was performed with standard procedures. We used two types of lysate samples, glomerular lysates (Glo) isolated from C57BL/6 mouse glomeruli using Dynabead perfusion as described [36] and lysates from kidney that lacked glomerular fractions, the rest of kidney (ROK). The membrane loaded with two Glo lysates and two ROK lysates was used for Stag1, Tmem22, IL-20rb and β-actin. The membrane with single Glo lysate and ROK lysate was used for Nck1 and calnexin, an internal control.

### qPCR

Glomeruli from 9 adult mice (4 wild-type C57BL/6 mice and 5 wild-type mice with the C57BL/6×129/Sv background) were isolated as mentioned above. Total RNA was isolated from human cultured cells and mouse glomeruli (Qiagen). The first-strand cDNA synthesis was carried out according to the manufacturer’s protocol (Invitrogen). TaqMan probes were purchased (Applied Biosystems) and qPCR was performed using the ABI PRISM 7300 Sequence Detection System (Applied Biosystems). Triplicate for each sample was carried out. The relative quantification of gene expression was analyzed using the comparative threshold (Ct) method. Data was presented as mean ± s.e.m 2^-ΔCt^.

### Ethics Statement

Both transgene manipulation in zebrafish and studies in mice were approved by the local ethical committee (the North Stockholm district court). The mice were housed with regulated light and dark cycles under pathogen-free conditions at the Scheele Animal Facility (Karolinska Institutet) and they had access to food and water *ad libitum*. All diabetic patients who were recruited from Finland gave written, informed consent to participate in the study and the Ethical Committee of the Finnish National Public Institute approved the protocol for the study.

### Statistics

Data of luciferase assay and qPCR was statistically analyzed by *t*-test. The Fisher’s exact test was used for analysis of zebrafish GFP expression rate. The Fisher’s exact test and *t*-test were used for association analysis of clinical data with genotypes.
